# Neutrophil Infiltration Characterized by Upregulation of S100A8, S100A9, S100A12 and CXCR2 Is Associated With the Co-Occurrence of Crohn’s Disease and Peripheral Artery Disease

**DOI:** 10.3389/fimmu.2022.896645

**Published:** 2022-06-20

**Authors:** Ziping Yao, Bihui Zhang, Guochen Niu, Ziguang Yan, Xiaoqiang Tong, Yinghua Zou, Yuan Li, Min Yang

**Affiliations:** ^1^ Department of Interventional Radiology and Vascular Surgery, Peking University First Hospital, Beijing, China; ^2^ Department of Hematology, Peking University First Hospital, Beijing, China

**Keywords:** peripheral artery disease, Crohn’s disease, neutrophil, immune process, bioinformatics

## Abstract

**Background:**

Crohn’s disease (CD) and peripheral arterial disease (PAD) are closely related. The pathophysiological mechanisms underlying the coexistence of CD and PAD are unknown. The aim of this study was to investigate the key molecules and pathways mediating the co-occurrence of CD and PAD through quantitative bioinformatic analysis of a public RNA sequencing database.

**Methods:**

Datasets of CD (GSE111889) and PAD (GSE120642) were downloaded from the Gene Expression Omnibus (GEO) database. Differentially expressed genes (DEGs) were analyzed using the ‘edgeR’ and ‘limma’ packages of R. Gene Ontology and Kyoto Encyclopedia analyses of common DEGs were performed to explore the functions of DEGs. Protein–protein interaction (PPI) networks were established by the Search Tool for the Retrieval of Interacting Genes (STRING) database and visualized by Cytoscape. Hub genes were selected using the plugin cytoHubba. Hub gene validation was performed in GSE95095 for CD and GSE134431 for PAD. Receiver operating characteristic curves were used to evaluate the predictive values of the hub genes. Gene set enrichment analysis and immune infiltration of the hub genes were performed.

**Results:**

A total of 54 common DEGs (2 downregulated and 52 upregulated) were identified. Pathways of neutrophil chemotaxis, neutrophil migration and cytokine and cytokine receptors were enriched in CD and PAD. S100A8, S100A9, S100A12 and CXCR2 were identified as hub genes after validation, with all area under the curve > 0.7 for both CD and PAD. Neutrophil infiltration was associated with upregulation of the hub genes. Pathways of immune processes, including neutrophil activation, neutrophil chemotaxis, neutrophil migration were significantly correlated with high expression of S100A8, S100A9, S100A12 and CXCR2 in both CD and PAD.

**Conclusions:**

This bioinformatic study elucidates S100A8, S100A9, S100A12 and CXCR2 as hub genes for the co-occurrence of Crohn’s disease and peripheral artery disease. Inflammation and immune regulation modulated by neutrophil infiltration play a central role in the development of CD and PAD and may be potential targets for diagnosis and treatment.

## Introduction

Crohn’s disease (CD) is a form of inflammatory bowel disease (IBD) that refers to a group of chronic inflammatory diseases that mainly target the gastrointestinal tract (1). In recent years, the prevalence and incidence of CD have increased rapidly, especially in Asia (2, 3). CD is believed to be caused by multiple factors, including genetic susceptibility, environmental factors, and gut microbiota, resulting in an abnormal mucosal immune response and compromised epithelial barrier function (2). However, the etiopathogenesis has not been fully elucidated (1).

CD is a well-established risk factor for arterial diseases, including ischemic artery disease, stroke and mesenteric ischemia (4–7). Chronic inflammation, endothelial dysfunction and autoimmune responses are thought to be factors contributing to the acceleration of atherosclerosis in CD (1, 8). The gut microbiota has been considered associated with cardiovascular diseases in recent years, but the role of CD-induced gut microbiota alterations in cardiovascular diseases is largely unclear (9).

Peripheral artery disease (PAD) refers to a reduction in blood flow to the affected limb due to arterial stenosis or occlusion caused by atherosclerosis (10). Recent studies have shown that CD is also associated with a high risk of PAD and that disease activity is an independent risk factor (11, 12). PAD also increases the risk of CD, which indicates the common mechanism of PAD and CD (13). Considering the disparities between PAD and diseases of other artery territories, the mechanisms of concomitant PAD and CD may be specific (14). Due to the rapid growth of PAD and its importance as a cause of morbidity and mortality in CD patients, it is critical to explore the common mechanisms of PAD and CD and to establish the role of inflammation, immune regulation and gut microbiota in this regard (15, 16).

In the present study, we investigated the common hub genes of PAD and CD and estimated the pathways involved in the hub genes by quantitative bioinformatic analysis of a public RNA-sequencing database. The identification of common hub genes and pathways will help to elucidate the common mechanisms of PAD and CD.

## Methods

### Study Design and Data Collection

All eligible datasets were downloaded from the Gene Expression Omnibus (GEO) database. We searched for related gene expression datasets using Crohn’s disease and peripheral artery disease as keywords. The selection criteria were as follows: 1) The arrays contained at least 15 samples in each group for PAD and 50 samples in each group for CD; 2) The included test specimens were from adult humans. Based on the above selection criteria, 2 datasets were finally included in this study, GSE120642 (17) and GSE111889 (18). The GSE120642 dataset contains the RNA-sequencing results of gastrocnemius muscle from 15 healthy controls and 36 PAD samples. GSE111889 includes biopsy samples from 50 healthy controls and 126 CD patients for RNA sequencing analysis (**Supplementary Table 1**).

### Identification of Differentially Expressed Genes With R Software

We downloaded the series matrix files of datasets from GEO. For GSE111889, the data were normalized, and DEGs were found using the R package ‘edgeR’ (19). For GSE120642, the DEGs between healthy and PAD samples were identified using the ‘limma’ package. The fold changes (FCs) were calculated for individual gene expression. Genes meeting specific cutoff criteria of P<0.05 and |logFC|>1.0 were defined as DEGs. The online Venn diagram tool was used to obtain their common DEGs (http://bioinformatics.psb.ugent.be/webtools/Venn/).

### Functional Annotation and Pathway Enrichment Analysis

To further reveal the functions of common DEGs, Gene Ontology (GO) annotation and Kyoto Encyclopedia of Genes and Genomes (KEGG) pathway enrichment analysis of DEGs were performed using the ‘cluster profiler’ package in R software (20). GO terms consist of the following three components: biological process (BP), cellular component (CC) and molecular function (MF).

GO functional enrichment analysis and KEGG pathway enrichment of the significant module were conducted using the Database for Annotation, Visualization, and Integrated Discovery (DAVID) (http://david.abcc.ncifcrf.gov/) online tool (21). A P value < 0.05 was set as the cutoff criterion.

### PPI Network Construction and Identification of Hub Genes

Based on the identified common DEGs, PPI networks were constructed using the Search Tool for the Retrieval of Interacting Genes Database (STRING) (https://cn.string-db.org/) and visualized by Cytoscape 3.9.0 (22). Confidence scores were set to intermediate values (confidence scores > 0.4). CytoHubba, a plug-in for Cytoscape software, was used to identify hub genes. CytoHubba is a plug-in for Cytoscape that measures nodes according to their network characteristics and therefore allows exploring important nodes in biological networks (23). In addition, MCODE, a plugin for Cytoscape, was used to filter the significant modules of core genes from the PPI network complex. The criteria were set as follows: Degree Cutoff = 2, Node Score Cutoff = 0.2, K-Core = 2, and Max. Depth = 100.

### Validation of Hub Gene Expression

The expression levels of the identified hub genes were validated in GSE95095 for CD and GSE134431 for PAD (24). GSE95095 contains 24 CD and 12 control samples. GSE134431 contained 8 samples of diabetic ulcers which were considered as PAD according to the GVG guideline (25), and 13 control samples. The comparison of the two datasets was performed using the Wilcoxon test. A P value < 0.05 was considered significant.

### Receiver Operating Characteristic Curves

Receiver operating characteristic (ROC) curves were generated to assess the predictive accuracy of the hub genes using the pROC package in the R language (26).

### Immune Infiltration Analysis

The ssGSEA (single-sample gene set enrichment analysis) algorithm is a rank-based method that defines a score representing the degree of absolute enrichment of a particular gene set in each sample (27, 28). The ssGSEA score was used to quantify the infiltration of immune cells in CD or PAD tissues and determine the level of immune infiltration in each sample of datasets. Correlations between 4 hub genes and 23 immune cells were determined using Spearman correlation analysis to reveal the relationship between hub genes and immune cells (29).

### Gene Set Enrichment Analysis

Gene expression levels were set as population phenotypes, and GSEA (http://software.broadinstitute.org/gsea/index.jsp) was used to assess related pathways and molecular mechanisms between the two groups (30). Enriched gene sets with nominal P values of < 0.05, |normalized enrichment scores (NES) | > 1 and false positive rate (FDR) q values of < 0.25 were considered significant.

### Statistical Analysis

In this study, all statistical analyses were performed using R software (version 4.1.2; https://www.r-project.org/). A value of P < 0.05 was considered significant.

## Results

### Identification of Common DEGs

**Figure 1:**
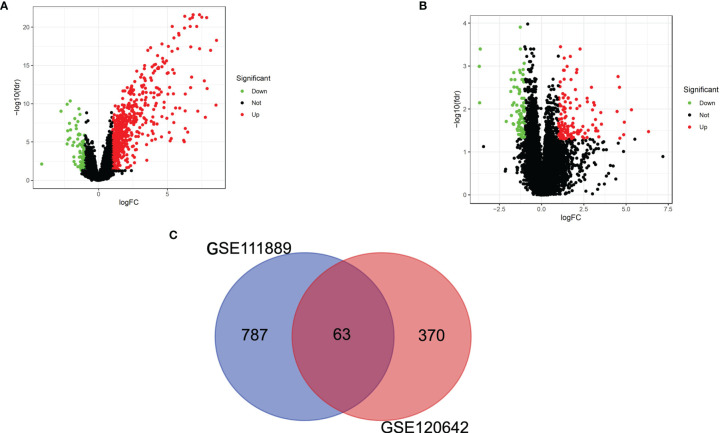
Identification of common DEGs. **(A)** Volcano plot revealing 850 DEGs between the CD patients and healthy controls. **(B)** Volcano plot revealing 433 DEGs between PAD patients and healthy controls. **(C)** A total of 63 common DEGs were identified after taking the intersection of DEGs in CD and PAD. DEG, differentially expressed gene; CD, Crohn’s disease; PAD, peripheral artery disease.

As shown in **Figure 1A**, 850 DEGs were screened between CD and healthy subjects using the edgeR package. There were 433 DEGs in PAD patients compared to healthy controls (**Figure 1B**). A total of 63 common DEGs were identified after taking the intersection of the Venn diagrams (**Figure 1C**). Heat maps of the intersecting DEGs in GSE120642 and GSE111889 are shown in **Supplementary Figure 1**. After excluding genes with opposite expression trends, 54 common DEGs with the same expression trends were found in GSE120642 and GSE111889, including 52 common upregulated genes and 2 common downregulated genes.

### GO and KEGG Pathway Analyses

**Figure 2:**
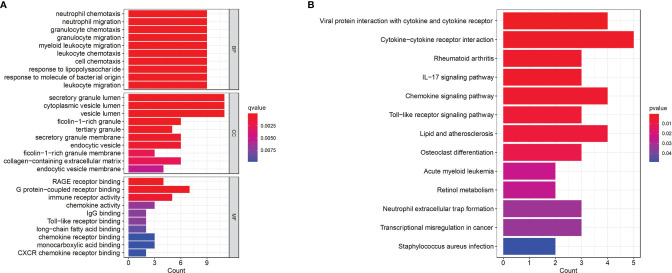
Functional enrichment analysis of the common DEGs. **(A)** GO terms in biological process, cellular component, and molecular function were used for functional enrichment clustering analysis on common DEGs. **(B)** KEGG pathway analysis was performed on common DEGs. DEG, differentially expressed genes; GO, Gene Ontology; KEGG, Kyoto Encyclopedia of Genes and Genomes.

To further explore the underlying biological information of these common DEGs, GO analysis and KEGG pathway enrichment were performed using the ‘cluster profiler’ package in R software. The results of these analyses showed that in terms of the biological process, the genes were mainly enriched in neutrophil chemotaxis, neutrophil migration and granulocyte chemotaxis. In terms of cellular components, the genes were mainly enriched in the secretory granule lumen, cytoplasmic vesicle lumen and vesicle lumen. Finally, in terms of molecular function, the genes were mainly enriched in RAGE receptor binding, G protein-coupled receptor binding and immune receptor activity (**Figure 2A**). In addition, KEGG analysis showed that DEGs were mainly enriched in viral protein interactions with cytokines and cytokine receptors, cytokine–cytokine interaction receptors and the IL-17 signaling pathway (**Figure 2B**).

### PPI Network Construction of Common DEGs

**Figure 3:**
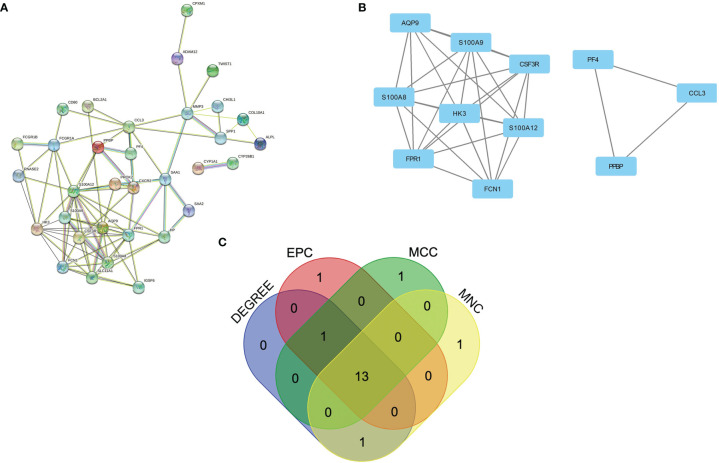
PPI network and identification of hub genes. **(A)** PPI network of the common DEGs constructed by STRING. **(B)** Significant gene module and enrichment analysis of the modular genes. **(C)** Identification of 13 candidates for hub genes by four algorithms. PPI, protein–protein interaction, DEG; differentially expressed genes; STRING, Search Tool for the Retrieval of Interacting Genes.

To further reveal the potential relationships between proteins encoded by common DEGs and identify hub genes, the PPI network of the DEGs was screened by STRING, including 50 nodes and 89 edges, with a PPI enrichment p value< 1.0e-16 (**Figure 3A**). MCODE, a plug-in of Cytoscape, was used to conduct module analysis to detect crucial clustering modules. Two modules were retrieved from the PPI network constructed using common DEGs. Module 1 included 8 nodes and 27 edges with a cluster score (density times the number of members) of 7.714 (**Figure 3B**). Module 2, with 3 nodes and 3 edges, had a score of 3 (**Figure 3B**).

GO analysis and KEGG pathway enrichment were performed for the most significant module to explore the underlying biological processes. From the results of GO analysis, the gene function of this module was associated with RAGE receptor binding and neutrophil chemotaxis (**Supplementary Table 2**). The KEGG pathway enrichment analysis showed that this module was mainly enriched in the IL-17 signaling pathway (**Supplementary Table 3**).

### Identification and Validation of Hub Genes

To explore genes that may play an important role in the co-occurrence of CD and PAD, hub genes for common DEGs were identified by CytoHubba. Since biological networks are heterogeneous, multiple topological analysis algorithms were used simultaneously to identify hub genes. MCC, MNC, Degree, and EPC were used to predict and explore the top 15 important hub genes in the PPI networks. The intersection of these 15 genes from the four algorithms revealed 13 candidate hub genes: HK3, S100A12, FCGR1A, S100A9, SLC11A1, FPR1, SAA1, S100A8, CXCR2, FCN1, AQP9, CSF3R, and HP (**Figure 3C**). The details of the candidate hub genes are shown in **Supplementary Table 4**.

**Figure 4:**
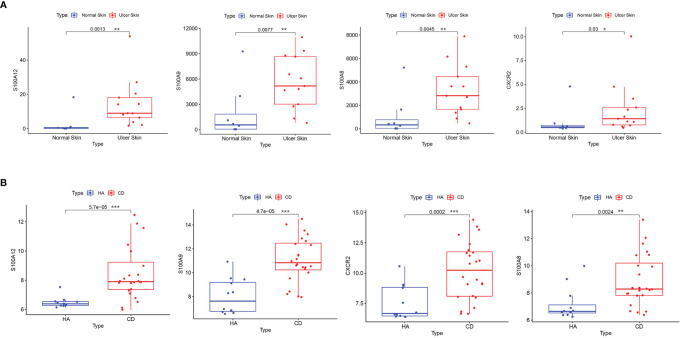
Validation of hub genes. **(A)** S100A8, S100A9, S100A12 and CXCR2 were validated in GSE134431. The boxplots show that the expression levels of the four hub genes are higher in ulcer skin samples. **(B)** S100A8, S100A9, S100A12 and CXCR2 were validated in GSE95095. The boxplots show that the expression levels of the four hub genes are higher in CD samples. *p < 0.05, **p < 0.01, ***p < 0.001. CD: Crohn’s disease.

Validation was performed in GSE95095 for CD and GSE134431 for PAD from GEO. The results showed that only four hub genes were significantly upregulated in both CD and PAD compared with normal tissues. The hub genes were S100A8, S100A9, S100A12 and CXCR2 (**Figure 4**).

### Efficacy Evaluation and PPI Construction of Hub Genes

**Figure 5:**
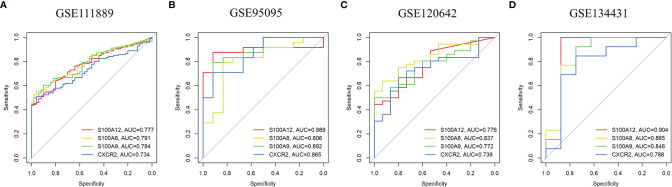
ROC curves of the hub genes in CD and PAD. ROC curves were drawn to evaluate the accuracy of the hub genes in diagnosing CD **(A, B)** or PAD **(C, D)**. ROC: receiver operating characteristic CD: Crohn's disease. PAD: peripheral artery disease.

Using the pROC package in R language, ROC curves were drawn in these four datasets with the expression of the four hub genes to assess the accuracy of the diagnostic features (**Figure 5**). The AUC values for all four genes were greater than 0.7 in all datasets including GSE95095 and GSE134431, demonstrating that the maximum plausible predictive capabilities of the four hub genes are excellent. Moreover, the ROC curves of the other 9 candidate genes in all datasets were shown in **Supplementary Figure 2**. These results indicated that S100A8, S100A9, S100A12 and CXCR2 can be promising markers for diagnosing PAD and CD.

**Figure 6:**
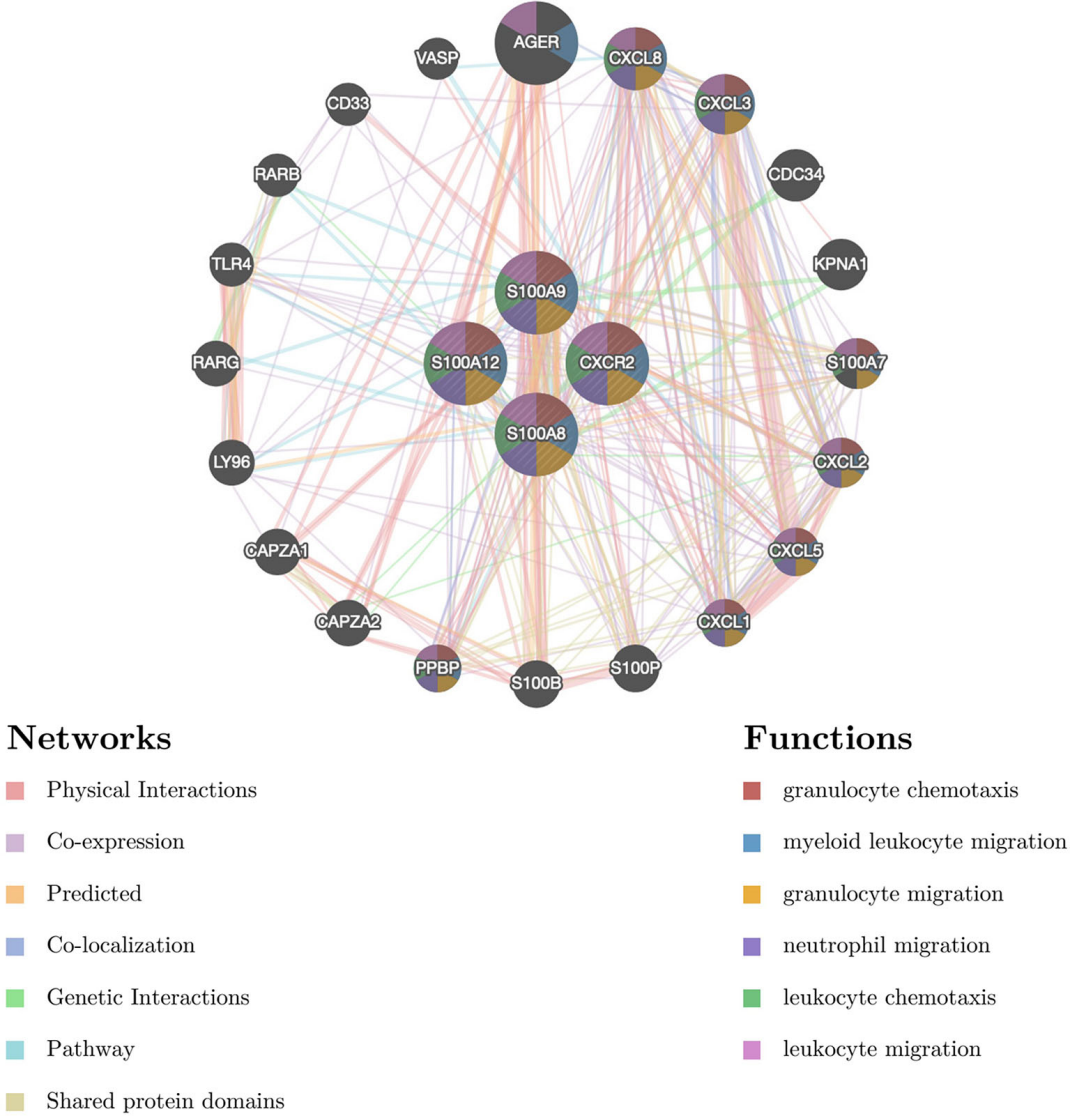
The gene–gene interaction network for DEGs was analyzed using the GeneMANIA database. The 20 most frequently changed neighboring genes are shown. The predicted genes are located in the outer circle, and hub genes are in the inner circle DEG: differentially expressed gene.

PPI analysis of the four hub genes and their 20 interacting genes was performed by GeneMANIA to predict correlations among colocalization, shared protein domains, coexpression, prediction and pathways (**Figure 6**). The predicted genes are located in the outer circle, while hub genes are in the inner circle. As shown in **Figure 6**, the network illustrates that these genes are enriched in granulocyte chemotaxis, myeloid leukocyte migration, granulocyte migration, neutrophile migration, leukocyte chemotaxis, and leukocyte migration.

### Association Between the Hub Genes and Immune Infiltration

**Figure 7:**
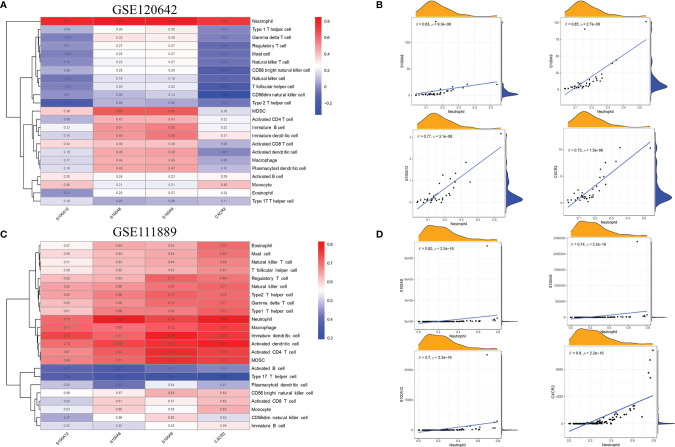
Association between the hub genes and immune infiltration. **(A)** In GSE120642, the level of neutrophil infiltration was significantly associated withhub genes in PAD samples. Red: positive correlation; blue: negative correlation. **(B)** Detailed correlation results of hub genes and neutrophil infiltration in GSE120642. **(C)** In GSE111889, S100A8, S100A9, S100A12 and CXCR2 were shown to positivelycorrelate with most cell types except for activated B cells, plasmacytoid dendritic cells,CD56dim natural killer cells, CD56 bright natural killer cells and Type 17 T helper cells.Red: positive correlation; blue: negative correlation. **(D)** Detailed correlation results of hubgenes and neutrophil infiltration in GSE111889. PAD: peripheral artery disease. CD: Crohn's disease.

The relationship between the 4 hub genes and 23 immune cells was analyzed by Spearman’s correlation analysis based on the results of GeneMANIA analysis of biological functions. As shown in **Figure 7A**, for PAD samples of GSE120642, the level of neutrophil infiltration was significantly associated with S100A8, S100A9, S100A12 and CXCR2. In addition, S100A8 and S100A9 were correlated with B cells, MDSCs, T cells and dendritic cells. In detail, all hub genes showed remarkable correlation with neutrophil cells with r > 0.7 and p < 0.05 (S100A8, r = 0.83, p = 9.3e-08; S100A9, r = 0.85, p = 2.7e-08; S100A12, r = 0.77, p = 3.1e-08; CXCR2: r = 0.73, p = 1.5e-06, **Figure 7B**). For CD samples in GSE111889, S100A8, S100A9, S100A12 and CXCR2 were positively correlated with most cell types, except for activated B cells, plasmacytoid dendritic cells, CD56dim natural killer cells, CD56 bright natural killer cells and Type 17 T helper cells (**Figure 7C**). Specifically, all hub genes showed remarkable correlation with neutrophil cells with r ≥ 0.7 and p < 0.05 (S100A8, r = 0.82, p < 2.2e-16; S100A9, r = 0.74, p < 2.2e-16; S100A12, r= 0.7, p < 2.2e-16; CXCR2: r = 0.8, p = 1.5e-06, **Figure 7D**). The correlation patterns were similar in validation databases of GSE95095 and GSE134431, as is seen in **Supplementary Figure 3**.

### GSEA Results of Hub Genes

**Figure 8:**
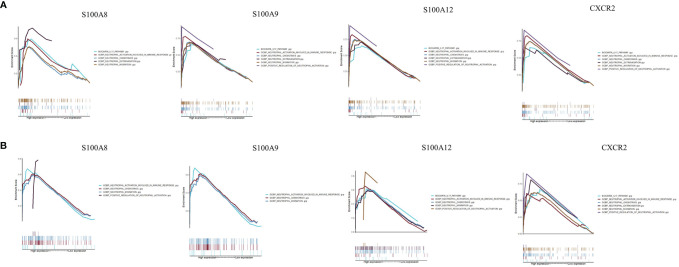
Gene set enrichment analysis. **(A)** A merged enrichment plot of S100A8, S100A9, S10012 and CXCR2 from gene set enrichment analysis in GSE120642, including enrichment score and gene sets. **(B)** A merged enrichment plot of S100A8, S100A9, S10012 and CXCR2 from gene set enrichment analysis in GSE111889, including enrichment score and gene sets.

To determine the potential functions of the hub genes on neutrophil cells in GSE120642 and GSE111889, GSEA was performed to identify differentially regulated pathways between the high and low expression groups of hub genes and to identify the activated signaling pathways in PAD and CD. GSEA analysis was performed for the gene sets ‘GOBP_POSITIVE_REGULATION_OF_NEUTROPHIL_ACTIVATION’, ‘GOBP_NEUTROPHIL_EXTRAVASATION’,’GOBP_NEUTROPHIL_ACTIVATION_INVOLVED_IN_IMMUNE_RESPONSE’,’GOBP_NEUTROPHIL_CHEMOTAXIS’, ‘GOBP_NEUTROPHIL_MIGRATION’, and ‘BIOCARTA_IL17_PATHWAY’. Activation of pathways including neutrophil activation, neutrophil chemotaxis, neutrophil migration was closely correlated with higher expression of S100A8, S100A9, S10012 and CXCR2 in GSE120642 (**Figure 8A**) and GSE111889 (**Figure 8B**). The nominal P values, NES and FDR q values for GSE120642 and GSE111889 were provided in **Supplementary Tables 5**, **6**.

## Discussion

In this study, common hub genes and pathways for CD and PAD were explored in a public database through bioinformatic analysis. S100A8, S100A9, S100A12 and CXCR2 were significantly upregulated in both CD and PAD patients and identified as hub genes. They showed the most remarkable correlation with neutrophil cells. GSEA analysis further indicates multiple immune responses including neutrophil activation, neutrophil chemotaxis, neutrophil extravasation, neutrophil migration was closely correlated with higher expression of those 4 hub genes. We therefore conclude S100A9, S100A8, S100A12, CXCR2 might play important roles in immune processes *via* affecting the activity of neutrophil cells during the progression of PAD and CD.

S100A8, S100A9, and S100A12 belong to the S100 family of EF-hand calcium-binding proteins and are involved in a wide variety of intracellular and extracellular functions. They are primarily expressed and secreted by granulocytes such as neutrophils (31). S100A8 and S100A9 are often present as heterodimers (S100A8/A9, also known as calprotectin), whereas S100A12 (also known as calgranulin C) is present as a homodimer (32). S100A8 and S100A9 account for up to 40% of neutrophil cytosolic proteins, while close to 5% are S100A12 (32). During inflammation, S100A8/A9 and S100A12 are actively released and exert a critical role in modulating the inflammatory response by stimulating leukocyte recruitment and inducing cytokine secretion in IBD (33, 34). The binding of S100A8/A9 to toll-like receptor (TLR) 4 or receptor for advanced glycation end products (RAGE) triggers the multiple pathways that play vital roles in inflammation (34). S100A12 is a ligand for RAGE, TLR4 and CD36, promoting cellular and immunological pathways to alter inflammation (31). High concentrations of S100A8/A9 and S100A12 are present in the serum and at inflammatory sites in patients with inflammation, so they can also be biomarkers of IBD diagnosis and disease activity (2, 33–35). S100A8, S100A9, and S100A12 also mediate the vascular inflammatory process (36). In many studies, serum S100A12 levels were found to be elevated in patients with CAD (36). S100A12 decreased after 52 weeks of a rigorous cardiovascular disease risk reduction program with comprehensive lifestyle changes (37). Patients with elevated S100A8/A9 had a significantly increased risk of death and myocardial infarction (38). In another study, plasma S100A12 was found to be a significant independent predictor of major adverse cardiovascular events in 652 patients with stable CAD who underwent percutaneous intervention (39). The Rotterdam study, a population-based cohort study that followed 839 participants without CAD for 10.6 years, found that S100A12 was the only biomarker among 16 other measured biomarkers of inflammation that was significantly associated with CAD after adjustment for age and sex (40). In patients undergoing hemodialysis, plasma S100A12 levels were strongly associated with the prevalence of PAD (41). In the present study, S100A8, S100A9 and S100A12 were identified as hub genes for CD and PAD. Immune processes, including granulocyte activation, granulocyte chemotaxis, neutrophil activation involved in the immune response, neutrophil chemotaxis, neutrophil migration and lymphocyte differentiation, play an important role in the development of CD and PAD.

CXCR2 is a G-protein coupled receptor (GPCR) responsible for cellular signal transduction (42). In neutrophils, CXCR2 is activated by chemokine CXCL8 (interleukin-8, IL-8) and regulates the recruitment of neutrophils from blood to sites of inflammation (43). In addition, CXCR2 is expressed by other cell types associated with chronic inflammation, including macrophages, lymphocytes, mast cells, dendritic cells and endothelial cells (43). Blocking CXCR2 signaling could be a potential therapeutic target for the prevention of IBD (42). CXCR2 has also been found to be associated with the progression of atherosclerosis (44). IL-8 interacts with CXCR2 on neutrophils, leading to the formation of neutrophil extracellular traps *via* Src and ERK and p38 MAPK signaling, aggravating the progression of atherosclerosis *in vivo (* 45). CXCR2 was identified as a common hub gene for CD and PAD. The recruitment of neutrophils and other immune cells is probably a common mechanism for CD and PAD.

Recent studies have shown that the gut microbiota is associated with cardiovascular diseases (9). Alterations in gut microbiota composition can determine the severity of myocardial infarction in rats (46). In addition, the metabolic potential of gut microbiota has been identified as a contributing factor in the development of cardiovascular diseases. Trimethylamine N-oxide (TMAO), which is the hepatic oxidation product of the microbial metabolite trimethylamine (TMA), is considered a potential promoter of atherosclerosis and cardiometabolic diseases (47). TMAO predicts long-term adverse event risk and incremental prognostic value in patients with PAD (48). However, although abnormal microbial composition is also found in IBD, it was found that TMAO was lower in patients with IBD than controls, which was a cardiovascular protective factor (49). Therefore, the role of gut microbiota in IBD may be achieved by modulating inflammatory and immune activity rather than metabolic products such as TMAO (50). In the present study, no genes associated with metabolic products of gut microbiota were identified, which supports the above theory.

The study had several limitations. First, the key pathways and hub genes identified in the present study have not been validated in laboratory experiments. However, the roles of S100A8, S100A9 and S100A12 have been elucidated in CD and PAD in multiple preclinical and clinical studies, which reflects the reliability of this study. Second, common hub genes of CD and PAD were validated in patients with CD or PAD, not with both diseases. The lack of a dataset including patients with both CD and PAD made the validation unattainable at the present time. This kind of validation should be performed in the future. Third, granulocytes are almost completely matured in the bone marrow with little gene expression occurring subsequently in tissue. As a result, potentially important key molecules of granulocytes may be missed or underrepresented in RNA sequencing of samples from other tissues.

## Conclusions

This bioinformatic study elucidates S100A8, S100A9, S100A12 and CXCR2 as common hub genes in CD and PAD. Inflammation and immune regulation modulated by neutrophil infiltration play a central role in the development of CD and PAD and may be potential targets for diagnosis and treatment.

## Data Availability Statement

The original contributions presented in the study are included in the article/**Supplementary Material**. Further inquiries can be directed to the corresponding authors.

## Author Contributions

ZPY and BZ contributed to the data analysis and writing. GN and ZGY contributed to data collection. YZ and XT contributed to the study supervision. BZ, YL and MY contributed to the study design. All authors contributed to the article and approved the submitted version.

## Funding

This work was supported by the Beijing Natural Science Foundation (grant No.7202203), National Natural Science Foundation of China (grant No.81970410), the Scientific Research Seed Fund of Peking University First Hospital (grant No. 2018SF023), the Youth Clinical Research Project of Peking University First Hospital (grant No. 2018CR16), and the Interdisciplinary Clinical Research Project of Peking University First Hospital (grant No. 2018CR33).

## Conflict of Interest

The authors declare that the research was conducted in the absence of any commercial or financial relationships that could be construed as a potential conflict of interest.

## Publisher’s Note

All claims expressed in this article are solely those of the authors and do not necessarily represent those of their affiliated organizations, or those of the publisher, the editors and the reviewers. Any product that may be evaluated in this article, or claim that may be made by its manufacturer, is not guaranteed or endorsed by the publisher.
