# Supplementary figures and images for: Neutrophil Infiltration Characterized by Upregulation of S100A8, S100A9, S100A12 and CXCR2 Is Associated With the Co-Occurrence of Crohn’s Disease and Peripheral Artery Disease
Source: Front Immunol. 2022 Jun 20;13:896645. doi: 10.3389/fimmu.2022.896645 (PMC9251382; doi:10.3389/fimmu.2022.896645)

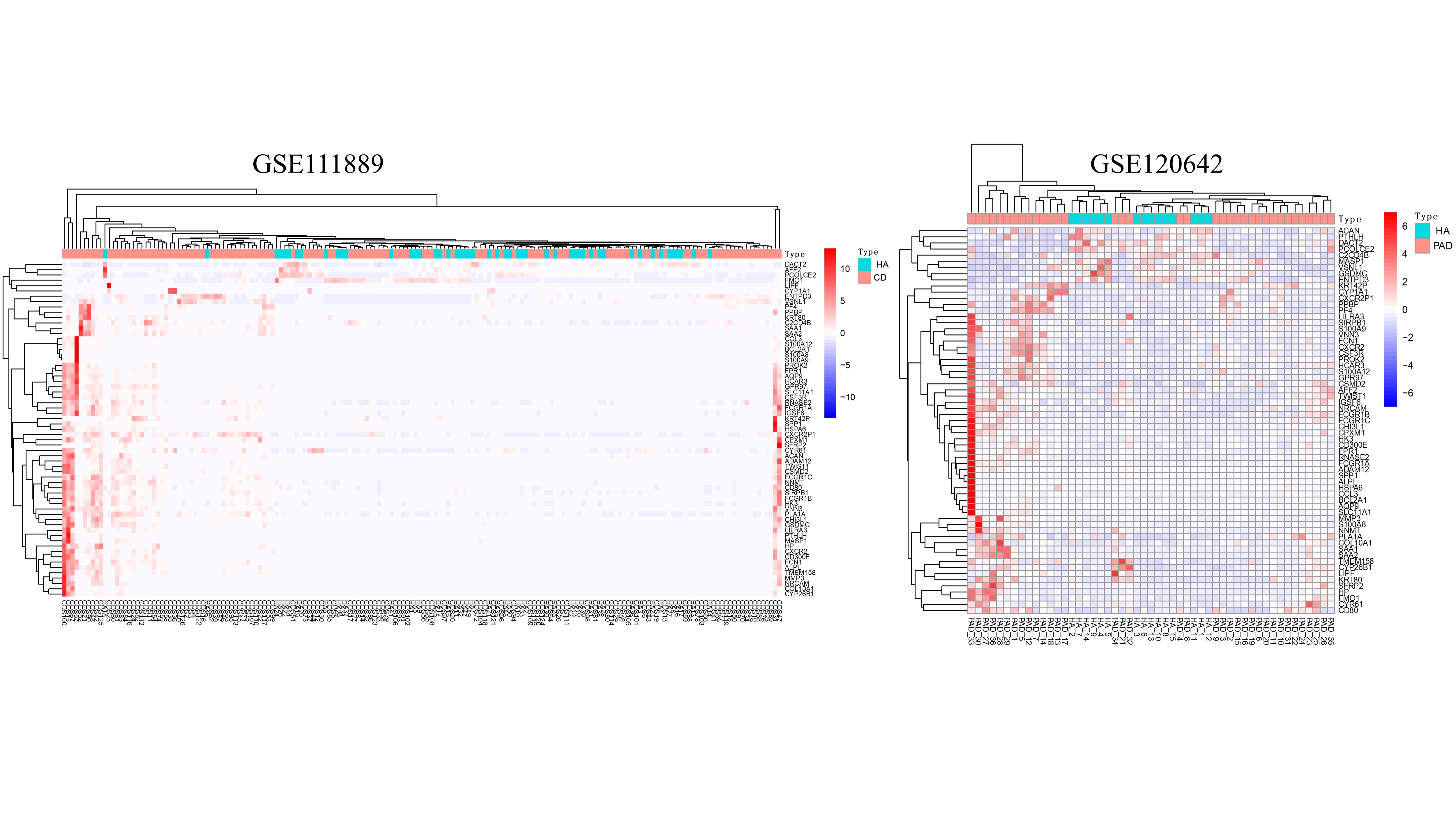

Supplement: Supplementary file 1 [file Image_1.jpeg]

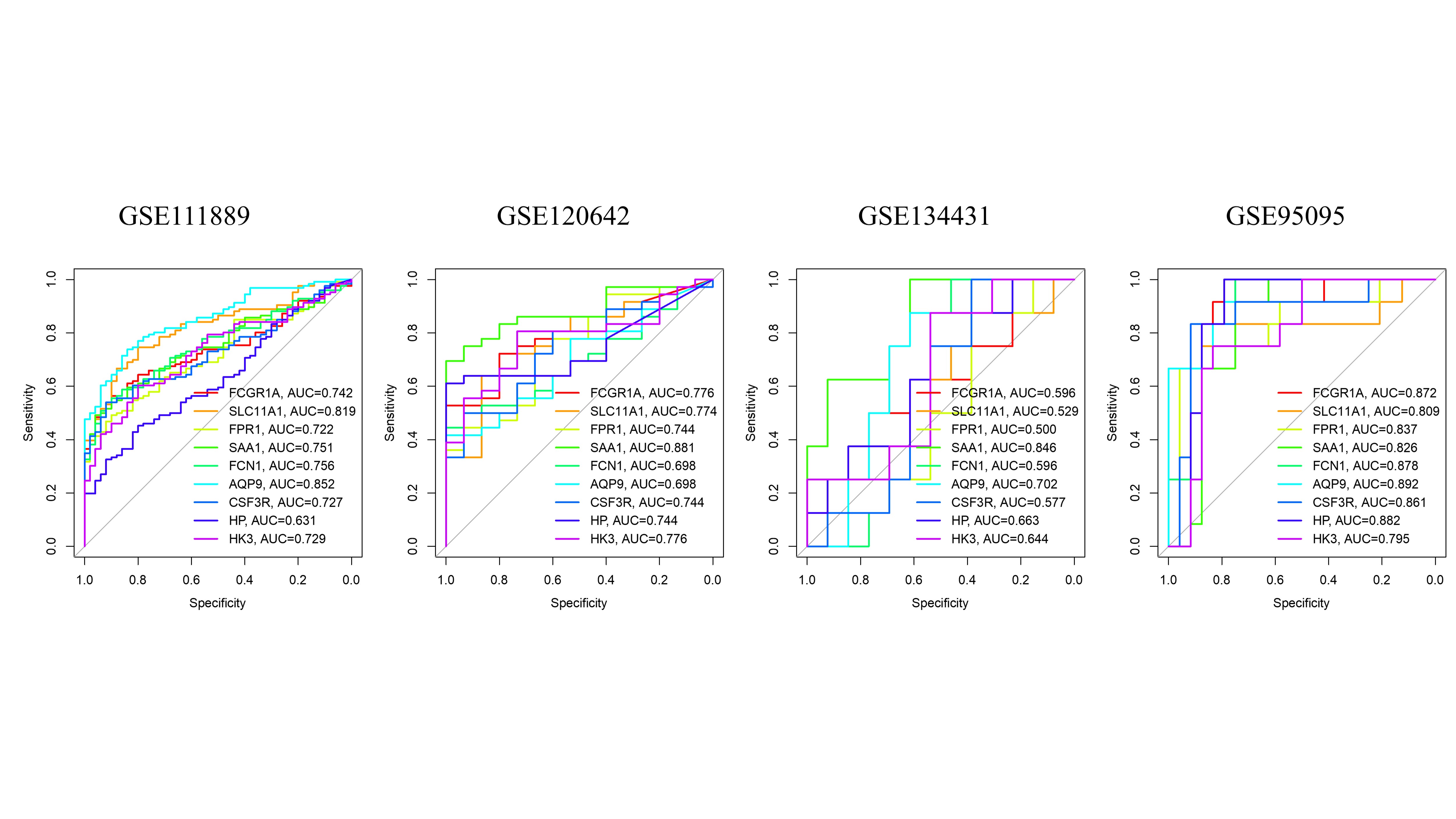

Supplement: Supplementary file 2 [file Image_2.jpeg]

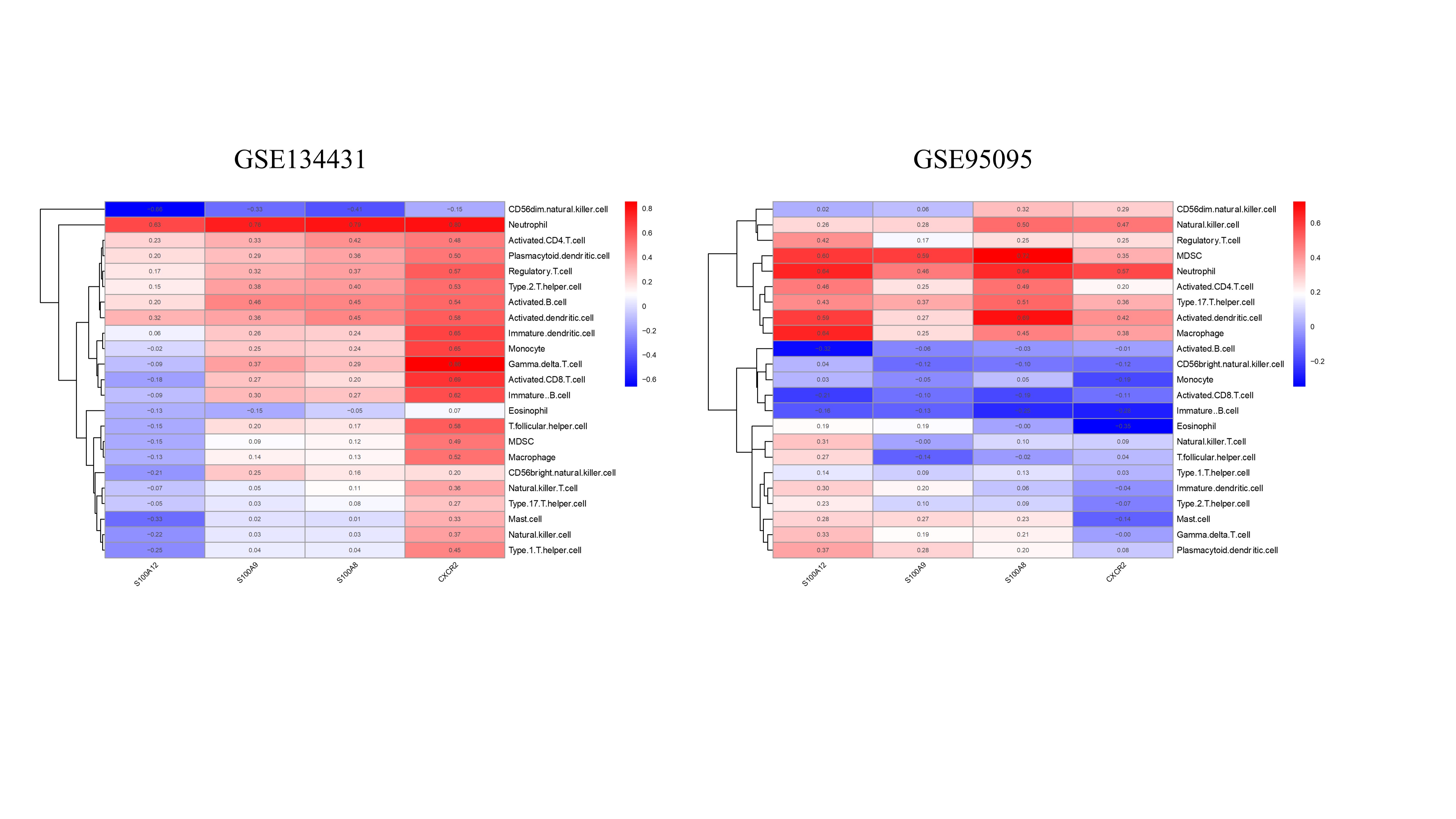

Supplement: Supplementary file 3 [file Image_3.jpeg]
