# Supplementary material for: Neutrophil Infiltration Characterized by Upregulation of S100A8, S100A9, S100A12 and CXCR2 Is Associated With the Co-Occurrence of Crohn’s Disease and Peripheral Artery Disease
Source: Front Immunol. 2022 Jun 20;13:896645. doi: 10.3389/fimmu.2022.896645 (PMC9251382; doi:10.3389/fimmu.2022.896645)
Supplement: Supplementary file 4 [file Table_1.docx]

Table 1. Number of samples and genes in datasets in this study.

| Data sets | Number of genes | Healthy samples | Patients’ samples |
| --- | --- | --- | --- |
| GSE120642 | 25343 | 15 | 36 PAD samples |
| GSE111889 | 21374 | 50 | 126 CD samples |
| GSE134431 | 27971 | 8 | 13 PAD samples |
| GSE95095 | 19875 | 12 | 24 CD samples |

Table 2. Enrichment analysis of GO enrichment of module 1.

| Category | Term | Count | PValue | FDR |
| --- | --- | --- | --- | --- |
| GOTERM_MF_DIRECT | GO:0050786~RAGE receptor binding | 4 | 5.23E-09 | 2.04E-07 |
| GOTERM_MF_DIRECT | GO:0048306~calcium-dependent protein binding | 3 | 4.91E-04 | 0.009577 |
| GOTERM_CC_DIRECT | GO:0034774~secretory granule lumen | 5 | 3.47E-08 | 9.71E-07 |
| GOTERM_CC_DIRECT | GO:0005576~extracellular region | 6 | 2.22E-04 | 0.00311167 |
| GOTERM_BP_DIRECT | GO:0043312~neutrophil degranulation | 6 | 1.89E-07 | 1.42E-05 |
| GOTERM_BP_DIRECT | GO:0030593~neutrophil chemotaxis | 4 | 2.44E-06 | 9.17E-05 |

Table 3. Enrichment analysis of KEGG pathway of module 1

| Category | Term | count | pValue | FDR |
| --- | --- | --- | --- | --- |
| KEGG_PATHWAY | hsa04657:IL-17 signaling pathway | 2 | 0.0566252 | 1 |

Table 4. The details of candidate hub genes

| Gene | Full name | Function |
| --- | --- | --- |
| HK3 | hexokinase 3 | Catalyzes the phosphorylation of hexose to hexose 6-phosphate |
| S100A12 | S100 calcium binding protein A12 | a calcium-, zinc- and copper-binding protein which plays a prominent role in the regulation of inflammatory processes and immune response |
| FCGR1A | Fc Gamma Receptor Ia | High affinity receptor for the Fc region of immunoglobulins gamma. Functions in both innate and adaptive immune responses. |
| S100A9 | S100 Calcium Binding Protein A9 | a calcium- and zinc-binding protein which plays a prominent role in the regulation of inflammatory processes and immune response |
| SLC11A1 | Solute Carrier Family 11 Member 1 | Divalent transition metal (iron and manganese) transporter involved in iron metabolism and host resistance to certain pathogens |
| FPR1 | Formyl Peptide Receptor 1 | High affinity receptor for N-formyl-methionyl peptides (fMLP), which are powerful neutrophil chemotactic factors |
| SAA1 | Serum Amyloid A1 | encodes a member of the serum amyloid A family of apolipoproteins |
| S100A8 | S100 Calcium Binding Protein A8 | a calcium- and zinc-binding protein which plays a prominent role in the regulation of inflammatory processes and immune response. |
| CXCR2 | C-X-C Motif Chemokine Receptor 2 | Receptor for interleukin-8 which is a powerful neutrophil chemotactic factor. Binding of IL-8 to the receptor causes activation of neutrophils |
| FCN1 | Ficolin 1 | Extracellular lectin functioning as a pattern-recognition receptor in innate immunity |
| AQP9 | Aquaporin 9 | Forms a water channel with a broad specificity. Also permeable glycerol and urea. |
| CSF3R | Colony Stimulating Factor 3 Receptor | Receptor for granulocyte colony-stimulating factor (CSF3), essential for granulocytic maturation |
| HP | Haptoglobin | As a result of hemolysis, hemoglobin is found to accumulate in the kidney and is secreted in the urine |

Table 5 The p values, q values and normalized enrichment scores (NES) of neutrophil-related gene-sets in GSE120642

| Gene-sets | S100A8 | | | S100A9 | | | S100A12 | | | CXCR2 | | |
| --- | --- | --- | --- | --- | --- | --- | --- | --- | --- | --- | --- | --- |
|  | p-val | FDR q-val | NES | p-val | FDR q-val | NES | p-val | FDR q-val | NES | p-val | FDR q-val | NES |
| GOBP_POSITIVE_REGULATION_OF_NEUTROPHIL_ACTIVATION | 0.063 | 0.043 | 1.54 | 0.004 | 0.003 | 2.00 | 0.000 | 0.002 | 1.88 | 0.005 | 0.012 | 1.70 |
| GOBP_NEUTROPHIL_EXTRAVASATION | 0.002 | 0.000 | 2.23 | 0.000 | 0.001 | 2.10 | 0.012 | 0.006 | 1.76 | 0.044 | 0.021 | 1.61 |
| GOBP_NEUTROPHIL_ACTIVATION_INVOLVED_IN_IMMUNE_RESPONSE | 0.000 | 0.000 | 2.14 | 0.000 | 0.000 | 2.77 | 0.000 | 0.000 | 2.31 | 0.000 | 0.000 | 2.29 |
| GOBP_NEUTROPHIL_CHEMOTAXIS | 0.000 | 0.000 | 2.79 | 0.000 | 0.000 | 3.66 | 0.000 | 0.000 | 2.60 | 0.000 | 0.000 | 2.59 |
| GOBP_NEUTROPHIL_MIGRATION | 0.000 | 0.000 | 2.92 | 0.000 | 0.000 | 3.84 | 0.000 | 0.000 | 2.60 | 0.000 | 0.000 | 2.57 |
| BIOCARTA_IL17_PATHWAY | 0.011 | 0.008 | 1.90 | 0.003 | 0.001 | 2.11 | 0.017 | 0.013 | 1.67 | 0.019 | 0.013 | 1.72 |

Table 6 The p values, q values and normalized enrichment scores (NES) of neutrophil-related gene-sets in GSE111889

| Gene-sets | S100A8 | | | S100A9 | | | S100A12 | | | CXCR2 | | |
| --- | --- | --- | --- | --- | --- | --- | --- | --- | --- | --- | --- | --- |
|  | p-val | FDR q-val | NES | p-val | FDR q-val | NES | p-val | FDR q-val | NES | p-val | FDR q-val | NES |
| GOBP_POSITIVE_REGULATION_OF_NEUTROPHIL_ACTIVATION | 0.089 | 0.088 | 1.43 | 0.048 | 0.054 | 1.5 | 0.014 | 0.015 | 1.65 | 0.002 | 0.002 | 1.76 |
| GOBP_NEUTROPHIL_EXTRAVASATION | 0.355 | 0.341 | 1.10 | 0.452 | 0.451 | 1.02 | 0.185 | 0.141 | 1.30 | 0.000 | 0.001 | 1.86 |
| GOBP_NEUTROPHIL_ACTIVATION_INVOLVED_IN_IMMUNE_RESPONSE | 0.000 | 0.002 | 1.78 | 0.005 | 0.002 | 1.85 | 0.005 | 0.005 | 1.79 | 0.006 | 0.004 | 1.69 |
| GOBP_NEUTROPHIL_CHEMOTAXIS | 0.000 | 0.000 | 2.21 | 0.000 | 0.000 | 2.19 | 0.000 | 0.000 | 2.26 | 0.000 | 0.000 | 2.15 |
| GOBP_NEUTROPHIL_MIGRATION | 0.000 | 0.000 | 2.20 | 0.000 | 0.000 | 2.18 | 0.000 | 0.000 | 2.26 | 0.000 | 0.000 | 2.10 |
| BIOCARTA_IL17_PATHWAY | 0.203 | 0.221 | 1.24 | 0.183 | 0.183 | 1.27 | 0.058 | 0.055 | 1.48 | 0.007 | 0.003 | 1.68 |

Table 7 The fold changes and p values of intersect differentially expressed genes (DEGs) in GSE120642 and GSE111889

|  | GSE111889 | | GSE120642 | |
| --- | --- | --- | --- | --- |
| ID | logFC | pValue | logFC | pValue |
| ACAN | 1.208906 | 0.000137 | -1.12868 | 0.008023 |
| ADAM12 | 1.452818 | 3.05E-06 | 2.691446 | 0.013137 |
| AFF2 | -1.73783 | 7.74E-11 | 1.266335 | 0.01471 |
| ALPL | 1.906938 | 1.03E-10 | 2.091458 | 0.010891 |
| AQP9 | 7.330379 | 2.00E-26 | 2.726543 | 0.027657 |
| BCL2A1 | 2.601549 | 2.95E-10 | 1.663533 | 0.019261 |
| C2CD4B | 1.13036 | 3.53E-08 | -1.06033 | 8.62E-05 |
| CCL3 | 3.973618 | 1.99E-17 | 3.216117 | 0.00119 |
| CD300E | 5.070002 | 2.57E-21 | 1.658515 | 0.002374 |
| CD80 | 1.003336 | 0.00013 | 1.773646 | 0.005435 |
| CHI3L1 | 4.93914 | 3.18E-19 | 4.859131 | 0.002294 |
| COL10A1 | 3.344582 | 7.94E-11 | 1.740937 | 0.006523 |
| CPXM1 | 1.779529 | 2.92E-10 | 3.061124 | 0.01646 |
| CSF3R | 3.344023 | 1.55E-18 | 1.517041 | 0.005625 |
| CSMD2 | 1.038612 | 5.19E-05 | 1.394636 | 0.000721 |
| CXCR2 | 4.606219 | 1.16E-21 | 1.504554 | 0.006899 |
| CXCR2P1 | 1.547864 | 2.96E-10 | 1.329626 | 0.033209 |
| CYP1A1 | 1.448526 | 0.00983 | 7.180885 | 0.024508 |
| CYP26B1 | 1.144611 | 7.51E-05 | 1.095032 | 0.003957 |
| CYR61 | 1.374489 | 1.67E-06 | 1.522915 | 0.000981 |
| DACT2 | -1.04171 | 2.19E-08 | -1.82295 | 2.70E-05 |
| ENTPD3 | 1.07157 | 2.66E-05 | -1.25244 | 2.71E-07 |
| FCGR1A | 2.383739 | 2.14E-15 | 2.180735 | 0.002114 |
| FCGR1B | 2.630103 | 3.26E-16 | 1.551721 | 0.017366 |
| FCGR1C | 2.37107 | 3.37E-11 | 2.871758 | 0.008491 |
| FCN1 | 3.321863 | 4.78E-18 | 1.17361 | 0.026549 |
| FMO1 | -1.1538 | 0.011877 | 1.796709 | 0.009454 |
| FPR1 | 4.287809 | 6.05E-20 | 1.711464 | 0.005625 |
| GPR97 | 1.965765 | 7.92E-11 | 2.153377 | 0.001506 |
| GSDMC | 2.766724 | 1.14E-10 | -1.79918 | 6.72E-06 |
| HCAR3 | 6.873554 | 2.21E-26 | 1.071215 | 0.047748 |
| HK3 | 1.604294 | 2.26E-11 | 1.444986 | 0.001598 |
| HP | 1.999923 | 3.79E-08 | 4.536872 | 0.006017 |
| HSPA6 | 1.441892 | 3.58E-09 | 3.121546 | 0.00525 |
| IGSF6 | 1.088724 | 6.27E-08 | 1.061272 | 0.031556 |
| KRT42P | 1.471588 | 0.00033 | 1.392901 | 0.001871 |
| KRT80 | 1.344948 | 1.94E-05 | 1.776289 | 0.00344 |
| LILRA3 | 3.847206 | 2.16E-16 | 1.736941 | 0.028977 |
| LIPF | 6.232874 | 2.11E-07 | 4.465525 | 0.000223 |
| MASP1 | 1.524424 | 2.06E-06 | -1.30579 | 2.29E-05 |
| MMP3 | 7.498947 | 9.30E-26 | 3.832931 | 0.012296 |
| NNMT | 1.517628 | 3.83E-09 | 2.42217 | 0.004563 |
| NRCAM | 1.140947 | 6.21E-05 | 1.429329 | 0.045465 |
| PCOLCE2 | -1.12116 | 1.68E-05 | -1.00565 | 0.00013 |
| PF4 | 1.727484 | 4.24E-08 | 3.025943 | 0.004086 |
| PLA1A | 1.364458 | 3.34E-09 | 1.025883 | 0.000981 |
| PPBP | 3.198247 | 6.66E-10 | 2.918714 | 0.01058 |
| PROK2 | 6.664081 | 3.74E-24 | 3.013803 | 0.000195 |
| PTHLH | 2.701501 | 2.32E-10 | -1.27788 | 0.000691 |
| RNASE2 | 1.830398 | 5.38E-10 | 5.31875 | 0.000184 |
| S100A12 | 8.569946 | 3.71E-22 | 2.573447 | 0.002002 |
| S100A8 | 7.854099 | 1.58E-25 | 3.087699 | 7.76E-05 |
| S100A9 | 7.13078 | 3.62E-24 | 2.088376 | 0.001871 |
| SAA1 | 5.340111 | 1.59E-20 | 4.524711 | 4.54E-06 |
| SAA2 | 5.060527 | 1.79E-17 | 4.608752 | 1.61E-05 |
| SFRP2 | 2.819077 | 6.62E-09 | 2.622223 | 0.004563 |
| SIRPB1 | 1.097395 | 0.000205 | 1.458279 | 0.00344 |
| SLC11A1 | 3.778872 | 4.32E-21 | 1.551818 | 0.00173 |
| SPP1 | 2.45722 | 3.96E-12 | 2.671239 | 0.024625 |
| TMEM158 | 1.517929 | 1.00E-08 | 1.015207 | 0.002544 |
| TWIST1 | 1.60661 | 7.79E-07 | 1.210396 | 0.02373 |
| VNN3 | 3.128694 | 1.21E-11 | 2.501806 | 0.030601 |
| VSNL1 | 1.292741 | 3.91E-05 | -1.2079 | 0.000286 |

Figure legends

Supplementary Figure 1. The heatmap of the intersect DEGs in GSE120642 and GSE111889.

Supplementary Figure 2. The ROC curves of the other 9 hub genes in four datasets.

Supplementary Figure 3. Association between the hub genes and immune infiltration in GSE95095 and GSE134431.
